# Trimethylation of Elongation Factor-Tu by the Dual Thermoregulated Methyltransferase EftM Does Not Impact Its Canonical Function in Translation

**DOI:** 10.1038/s41598-019-39331-x

**Published:** 2019-03-05

**Authors:** Samantha M. Prezioso, Duc M. Duong, Emily G. Kuiper, Qiudong Deng, Sebastián Albertí, Graeme L. Conn, Joanna B. Goldberg

**Affiliations:** 1 Microbiology and Molecular Genetics (MMG) Program, Graduate Division of Biological and Biomedical Sciences, Emory University, Atlanta, GA 30322 USA; 2 Division of Pulmonology, Allergy/Immunology, Cystic Fibrosis and Sleep, Department of Pediatrics, Emory University School of Medicine, Atlanta, GA 30322 USA; 3 Emory Integrated Proteomics Core, Emory University, Atlanta, GA 30322 USA; 4 Biochemistry, Cell and Developmental Biology (BCDB) Program, Graduate Division of Biological and Biomedical Sciences, Emory University, Atlanta, GA 30322 USA; 5 Department of Biochemistry, Emory University School of Medicine, Atlanta, GA 30322 USA; 6 Instituto Universitario de Investigación en Ciencias de la Salud, Universidad de las Islas Baleares, Palma de Mallorca, Spain; 7 Emory Antibiotic Resistance Center, Atlanta, GA 30322 USA; 8 Emory + Children’s Center for Cystic Fibrosis and Airway Disease Research, Atlanta, GA 30322 USA

## Abstract

The *Pseudomonas aeruginosa* methyltransferase EftM trimethylates elongation factor-Tu (EF-Tu) on lysine 5 to form a post-translational modification important for initial bacterial adherence to host epithelial cells. EftM methyltransferase activity is directly temperature regulated. The protein stability of EftM is tuned with a melting temperature (T_m_) around 37 °C such that the enzyme is stable and active at 25 °C, but is completely inactivated by protein unfolding at higher temperatures. This leads to higher observable levels of EF-Tu trimethylation at the lower temperature. Here we report an additional layer of thermoregulation resulting in lower *eftM* mRNA transcript level at 37 °C compared to 25 °C and show that this regulation occurs at the level of transcription initiation. To begin to define the impact of this system on *P*. *aeruginosa* physiology, we demonstrate that EF-Tu is the only observable substrate for EftM. Further, we interrogated the proteome of three different wild-type *P*. *aeruginosa* strains, their *eftM* mutants, and these mutants complemented with *eftM* and conclude that trimethylation of EF-Tu by EftM does not impact EF-Tu’s canonical function in translation. In addition to furthering our knowledge of this *Pseudomonas* virulence factor, this study provides an intriguing example of a protein with multiple layers of thermoregulation.

## Introduction

*Pseudomonas aeruginosa* is an important opportunistic pathogen that can thrive in a wide variety of environments and hosts^1^. Temperature change is one of the potential signals that cue the transition from the environment to the human host. In response to this change, *P*. *aeruginosa* strain PAO1 has been shown to modulate 6.4% of its transcriptome in the shift from 23 °C (ambient environmental temperature), to 37 °C (human body temperature)^2^. Most virulence factor thermoregulation occurs such that the output of the virulence factor is triggered or increased at 37 °C in response to a mammalian host^3^. However, not all bacterial hosts are warm-blooded, and further, increased production of a virulence factor may actually be counterproductive to the long-term success of a bacterium. Therefore, to fine-tune expression to the new environment, some bacterial virulence factors are decreased in expression at 37 °C. One example of this regulatory trend in *P*. *aeruginosa* is Piv (protease IV; PA4175). This protein shows lower expression at 37 °C than 25 °C, despite being well accepted as an important virulence factor during infection^4,5^. Thermoregulation resulting in reduced virulence factor expression and/or activity is important but understudied compared to the more typically observed up-regulation at 37 °C and down-regulation at 25 °C.

Another example of a *P*. *aeruginosa* virulence factor that is down-regulated upon transition to 37 °C is EftM, a *S*-adenosyl-L-methionine (SAM)-dependent methyltransferase that trimethylates elongation factor-Tu (EF-Tu) on lysine 5 (K5me^3^). In addition to its essential canonical role in delivering charged tRNAs to the ribosome during translation^6^, EF-Tu is found on the bacterial cell surface^7^. While this post-translational modification does not appear to alter the ability of EF-Tu to be surface-localized^8^, once in this subcellular location, K5me^3^-modified EF-Tu performs a moonlighting role that increases bacterial adherence to human epithelial cells^8^. Interestingly, EftM activity is post-translationally controlled through inherent protein instability; EftM is stably folded at lower temperature (25 °C) but unfolds and loses methyltransferase activity at elevated temperatures (37 °C). However, whether there are additional thermoregulatory mechanisms impacting EftM regulation is less clearly defined. Several studies have investigated global RNA changes in *P*. *aeruginosa* in response to temperature by a variety of techniques, including microarray^2,5^ and RNA-seq.^9^. These studies reported no information on *eftM* in response to temperature, most likely because the *eftM* transcript level is below the limit of detection using these approaches. This limitation thus necessitated a directed approach for investigating the impact of temperature on *eftM*. Here we demonstrate *eftM* thermoregulation directly though RT-qPCR and show that the steady state levels of *eftM* are higher at 25 °C than 37 °C. Further, using reporter fusions, we reveal that this additional layer of thermoregulation is controlled at the level of transcriptional initiation.

EftM’s only currently recognized cellular target for methylation is EF-Tu. EF-Tu is an extremely abundant protein in the cell during exponential phase growth, accounting for 6–13.5% of total cellular protein and outnumbering ribosomes 8–14 to one, depending on growth rate^10^. As mentioned, EF-Tu is an elongation factor that delivers charged tRNA to the ribosome during translation and contributes to proofreading of the growing peptide chain^11^. This canonical function is essential for bacterial cells^12^; however, post-translational modifications can alter this function. For example, in *Escherichia coli*, EF-Tu lysine 56 is methylated in response to bacterial energy state with monomethylation being observed during exponential phase growth and dimethylation being observed during nutrient limitation, such as in stationary phase. Lysine 56 methylation affects EF-Tu canonical function by slowing the rate of EF-Tu GTP hydrolysis and therefore reducing translation capacity^13^. In addition to methylation, EF-Tu can be phosphorylated, such as by the toxin Doc that phosphorylates EF-Tu threonine 382 in *E*. *coli*. This single post-translational modification is sufficient to completely halt translation through inactivation of *E*. *coli* EF-Tu^14^. EftM trimethylates EF-Tu on K5, found on the disordered loop at the protein amino-terminus. Given the essential nature of EF-Tu during translation and the impact post-translational modification of EF-Tu can have on its canonical role in protein synthesis, we aimed to uncover the impact of EF-Tu K5me^3^ on EF-Tu’s canonical function in translation. To do so, we utilized whole-cell proteomics to assess the proteome of three different *P*. *aeruginosa* strains in a label-free, unbiased manner to expose any effect K5me^3^ has on the global *Pseudomonas* proteomic landscape. These analyses reveal that methylation of EF-Tu by EftM has limited impact on the proteome under the conditions examined.

## Results

### *P*. *aeruginosa* has higher mRNA steady-state levels of *eftM* at 25 °C compared to 37 °C

**Figure 1:**
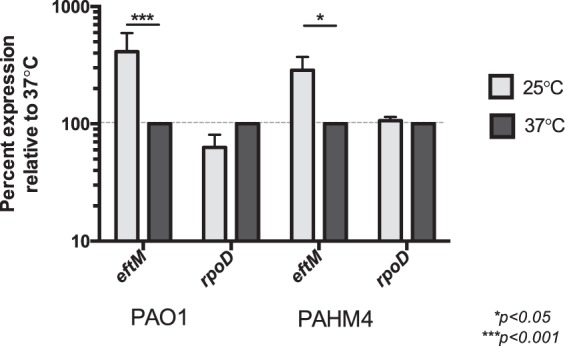
*P*. *aeruginosa* has higher mRNA steady-state levels of *eftM* at 25 °C compared to 37 °C. RT-qPCR was utilized to assess steady-state mRNA levels from mid-exponential cells grown at 25 °C compared to those grown at 37 °C. CT values for *eftM* were normalized to *omlA* as an internal control, then each temperature pair was compared by setting 37 °C as 100%. *rpoD* was evaluated in the same manner as a control for no change in response to temperature. Error bars represent standard deviation of the mean. Significance was determined by two-way ANOVA with Sidak multiple comparisons analysis. N = 3.

We have previously noted that in PAO1 the K5me^3^ modification of EF-Tu is more prominent at 25 °C compared to 37 °C^15^ and we showed that this thermoregulation was due, at least in part, to the unfolding of EftM at the higher temperature^16^. To determine whether another layer(s) of thermoregulation exists in addition to EftM protein stability, we first measured *eftM* mRNA levels by RT-qPCR. From this we observed an average of 48 copies of 25 °C *eftM* transcript per 1000 copies of our standard internal reference gene *omlA*, and only 11 copies of 37 °C *eftM* transcript per 1000 *omlA*. By comparing these steady state levels we conclude an average of 4X upregulation of PAO1 *eftM* mRNA transcripts at 25 °C compared to 37 °C (Fig. 1). In contrast for *rpoD*, used as a control transcript unaffected by temperature, similar levels were observed at both temperatures as expected^17^.

Chronic infection isolate PAHM4, originally isolated from non-CF bronchiectasis, encodes a mutated *mutS* and is therefore a hypermutator strain^18^. This defect in DNA mismatch-repair results in an ~1,000-fold increase in mutation rate and allowed for adaptation of the strain to the human lung. Interestingly, this strain shows a less extreme disparity between the levels of EF-Tu trimethylation at 25 °C vs 37 °C^8^. The amino acid sequence of EftM_PAHM4_ has 28 differences compared to the PAO1 protein. Ongoing studies have shown that these amino acid changes confer elevated thermostability such that the mid-point melting temperature (T_m_) of EftM from PAHM4 is 42 °C (unpublished observations) compared to ~36 °C PAO1 EftM^16^. To determine whether the *eftM* transcript is also impacted by temperature in PAHM4, we performed RT-qPCR analysis and found that steady state levels of PAHM4 *eftM* are about 3X higher at 25 °C compared to 37 °C. Again, no significant differences in relative expression of *rpoD* were observed (Fig. 1). These results indicate that, in addition to EftM being post-translationally thermoregulated through protein instability^16^, *eftM* is transcriptionally thermoregulated resulting in elevated mRNA levels at 25 °C. Importantly this result holds true for both PAO1 and PAHM4, indicating that the two layers of thermoregulation are not interlinked.

### Complementation of *eftM* mutants and maintenance of thermoregulation

**Figure 2:**
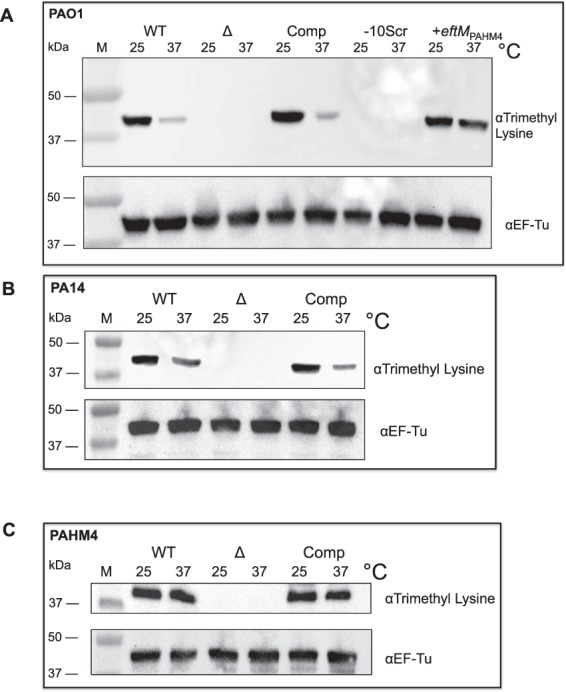
Survey of EftM activity in various strains. Western immunoblot with αTrimethyl Lysine (top) and αEF-Tu (bottom; loading control) for the following samples grown to mid-log at 25 °C and 37 °C: (**A**) PAO1 strain set. WT = PAO1, Δ = PAO1Δ*eftM*, Comp = PAO1Δ*eftM att*Tn7::P_PAO1_-*eftM*_PAO1_, −10Scr = PAO1Δ*eftM att*Tn7::P_PAO1-10SCR_-*eftM*_PAO1_, + *eftM*_PAHM4_ = PAO1ΔeftM *att*Tn7::P_HM4_-*eftM*_HM4_. See Fig. 3B for details of −10Scr complementation construct. (**B**) PA14 strain set. WT = PA14, Δ = PA14 *eftM*::tn, Comp = PA14 *eftM*::tn *att*Tn7:: P_PAO1_-*eftM*_PAO1_. (**C**) PAHM4 strain set. WT = PAHM4, Δ = PAHM4Δ*eftM*, Comp = PAHM4Δ*eftM att*Tn7::P_PAHM4_-*eftM*_PAHM4_.

We previously described the construction of a mutant, PAO1∆*eftM*, which is unable to modify EF-Tu. Interestingly, when this deletion strain was complemented with a multi-copy plasmid expressing *eftM* from a constitutive promoter, ablation of EF-Tu K5me^3^ temperature regulation was observed^15^. This was presumed to be due to overproduction of EftM and accumulation of EF-Tu methylation. To determine whether we could complement the *eftM* mutation while maintaining thermoregulation as observed in the wild-type strain, we transferred a single copy of *eftM* driven from its native promoter to an innocuous site of the *P*. *aeruginosa* chromosome of PAO1∆*eftM*. Wild-type PAO1 (WT), mutant PAO1∆*eftM* (∆), and the single-copy complemented PAO1∆*eftM* strain (Comp) were grown at 25 °C or 37 °C and assessed for trimethylation of EF-Tu by Western blotting with an αTrimethyl Lysine antibody. Antibody to EF-Tu served as an internal loading control. As seen in Fig. 2A (lower panel), the levels of EF-Tu are similar between all strains. As previously noted, WT shows greater trimethylation of EF-Tu at 25 °C compared to 37 °C, while ∆ shows no trimethylation at either temperature. Importantly, the level of trimethylation observed in the single-copy complement (Comp) was similarly temperature regulated compared to WT, indicating this construct reverses the original defect in the deletion mutant and resembles WT with respect to temperature regulation of EftM activity.

To confirm that the effect was not PAO1-specific, we performed similar single-copy native-promoter complementation and subsequent Western blot analysis with a PA14 transposon mutant, PA14 *eftM*::tn, and showed that both the WT and Comp of these strains showed similar thermoregulation of trimethylation of EF-Tu (Fig. 2B), while PA14 *eftM*::tn did not methylate EF-Tu at either temperature. We also constructed an *eftM* mutant in PAHM4 in the same manner as described for PAO1∆*eftM*. As seen in Fig. 2C, consistent with previous observations^8^, PAHM4 wild-type shows slightly more trimethylation of EF-Tu at 25 °C compared to 37 °C, while the newly constructed deletion mutant shows no trimethylation at either temperature. Complementing PAHM4∆*eftM* with *eftM*_PAHM4_ in single-copy with the native PAHM4 promoter restored expression to that seen in the parental strain (Fig. 2C).

We additionally transferred e*ftM*_PAHM4_ to PAO1Δ*eftM*. This set of PAO1 wild-type, deletion, PAO1-complemented *eftM*, and PAHM4-complemented *eftM* strains in one isogenic background allowed us to directly compare EftM with lower and higher T_m_s. When assessed by Western blotting, we noticed increased but not equal levels of methylation on EF-Tu at 37 °C compared to 25 °C for PAO1Δ*eftM* + *eftM*_PAHM4_. The difference between EF-Tu trimethylation in the *eftM*_PAO1_-complemented strain and the *eftM*_PAHM4_-complemented PAO1 strain (Fig. 2A) tells us that all information necessary for thermoregulation can be transferred with the upstream intergenic region and coding sequence for *eftM* and is not dependent on genomic context of *eftM* in the chromosome.

### Transcription of *eftM* is initiated from the same promoter at 25 °C and 37 °C

**Figure 3:**
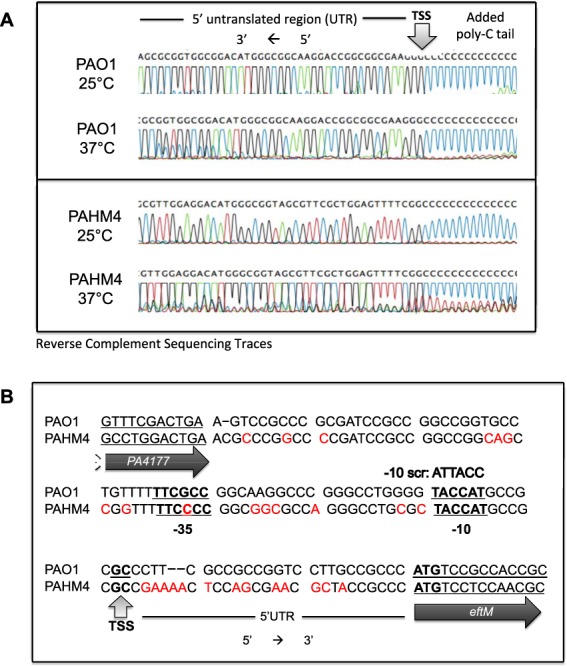
Mapping of *eftM* transcription start site and prediction of the promoter. (**A**) Sequencing trace files. 5′RACE was utilized to define the transcription start site for *eftM* in PAO1 and PAHM4 at 25 °C and 37 °C, with the same transcription start site (grey arrow) observed under both temperatures. Traces are the reverse complement of the 5′ untranslated region (UTR), with the transcription start site defined as the point of junction with the assay-added poly-nucleotide tail. (**B**) Sequences of the *eftM* intergenic region for PAO1 and PAHM4 are aligned with differences in red. Transcription Start Site (TSS) revealed in (**A**) is annotated, and the predicted −10 and −35 underlined. The sequence for the scrambled promoter strain (−10 Scr; PAO1Δ*eftM att*Tn7::P_PAO1-10SCR_-*eftM*_PAO1_ FLAG) is detailed above the predicted −10 region.

A potential mechanism for *eftM* transcriptional thermoregulation could be the utilization of different promoters at different temperatures; we therefore next studied the transcriptional thermoregulation of *eftM* by mapping the promoter. To accomplish this we defined the transcription start site (TSS) for both PAO1 and PAHM4 grown at both 25 °C and 37 °C and used this information for promoter prediction analysis. Using 5′ rapid amplification of cDNA ends (5′RACE) with mRNA harvested from cells grown at either 25 °C or 37 °C, we found the same TSS 26–27 nucleotides upstream of the translation start codon (ATG) in both strains (Fig. 3A).

Using the transcription start site as a guide we predicted *eftM*’s promoter to be (−35) TTCGCC and (−10) TACCAT (Fig. 3B). Despite differences between PAO1 and PAHM4 in the *eftM* upstream region, this prediction is conserved with one G→C polymorphism in the −35. To confirm this promoter prediction, we mutated the native promoter of *eftM* so that the −10 region was scrambled from TACCAT to ATTACC. This construct was then transferred to PAO1∆*eftM*. Unlike the construct with the wild-type sequence (Comp), this strain (−10Scr) showed no trimethylation of EF-Tu at either temperature (Fig. 2A). This result indicates that there is no transcription of *eftM* and thus proper identification of the promoter (Fig. 3B).

### Thermoregulation is at the level of transcription initiation

**Figure 4:**
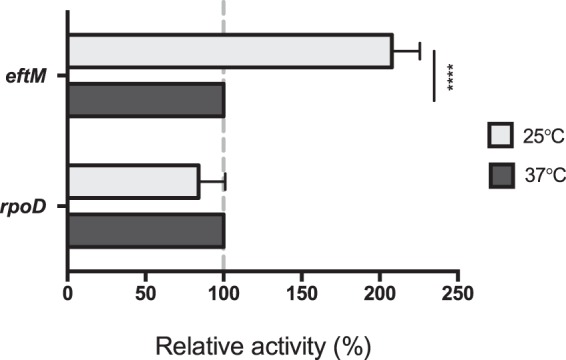
Thermoregulation is at the level of transcription initiation. β-galactosidase assay of the P*eftM* reporter strain (PAO1 *att*CTX::P_*eftM*_-*lacZ*) grown to mid-exponential phase at either 25 °C or 37 °C. Miller units of each 25 °C sample were normalized as a percent reactivity of the corresponding sample grown at 37 °C. P*rpoD* reporter strain (PAO1 *att*CTX::P_*rpoD*_-*lacZ*) was used as a control. Error bars represent standard deviation of the mean; significance was determined by two-way ANOVA with Sidak multiple comparisons analysis. N = 3. ****p < *0*.*0001*.

RT-qPCR revealed *eftM* mRNA steady state levels are higher at 25 °C than 37 °C (Fig. 1). Steady-state mRNA levels are modulated by a variety of factors, including transcription initiation and mRNA stability/decay. While 5′ RACE implied the same promoter was driving transcription at both temperatures (Fig. 3), this does not eliminate the possibility that transcription factors are altering transcription initiation in response to temperature. To investigate transcription initiation as the point of transcriptional thermoregulation, the entire 99-nucleotide *eftM* intergenic region, including the native *eftM* promoter and ribosome binding site, were fused seamlessly to a promoterless *lacZ* cassette and inserted in single copy to the PAO1 CTX attachment site^19^. An *rpoD* reporter was constructed and again utilized as a control for no change in response to temperature. Both reporter strains were probed by standard β-galactosidase assay for levels of transcription initiation, as proxied by LacZ activity, at 25 °C compared to 37 °C (Fig. 4). Our *eftM* reporter strain showed an average of a 207% increase in transcription initiation at 25 °C compared to 37 °C, while the *rpoD* control showed no significant difference, indicating that transcription initiation may be a mechanism mediating *eftM* thermoregulation.

### EF-Tu is the only observable cellular substrate for EftM

Our previous analysis of EftM protein stability^16^, and the current work here, reveal EftM to be a dual thermoregulated methyltransferase that trimethylates EF-Tu robustly at 25 °C. Post-translational modifications are known to be able to impact EF-Tu’s canonical role in translation. However, to study the impact of EF-Tu lysine 5 trimethylation on *Pseudomonas* physiology, we first needed to confirm that EF-Tu is the only substrate of EftM, allowing us to more firmly attribute any phenotype observed to EftM-mediated methylation of EF-Tu.

**Figure 5:**
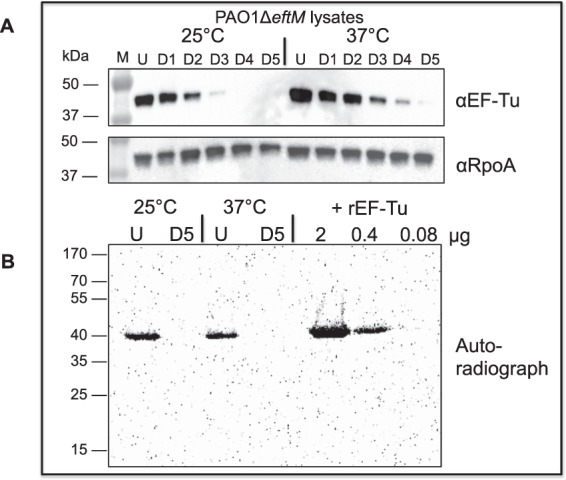
EF-Tu is the only observable substrate for EftM. (**A**) PAO1Δ*eftM* was grown to mid-exponential phase at 25 °C or 37 °C. The soluble lysate (U) from each sample was depleted for EF-Tu five subsequent times (D1-D5) by immunoprecipitation and analyzed by Western immunoblot using α-EF-Tu or α-RpoA (loading control). (**B**) 20 μg of undepleted (U) or fully depleted (D5) lysate was incubated with 3.6 μg purified EftM (8 μM final concentration) and [3 H]-SAM at 25 °C for 20 minutes and separated by SDS-PAGE. The gel was dried and exposed to a tritium screen overnight for the presence of proteins methylated by EftM. Recombinant EF-Tu (rEF-Tu) was added to D5 as a control for EftM enzyme activity and to indicate limit of detection.

Whole cell lysates of PAO1Δ*eftM* grown at both 25 °C and 37 °C were obtained and EF-Tu was removed from each using five rounds of immune-depletion. We first confirmed successful depletion of EF-Tu from the cellular lysates by probing for EF-Tu (Fig. 5A). We also probed for RpoA as a control to ensure that protein was not non-specifically being depleted. Next we performed *in vitro* methylation assays by adding recombinant, purified EftM with a radiolabeled form of its co-substrate SAM (adenosyl-L-methionine, S-[methyl-^3^H]) to detect other proteins receiving methyl-^3^H. Undepleted lysates (U) revealed methylation of a single protein, EF-Tu, at ~40 kDa as previously described^16^. In comparison, we observed no tritiated proteins between 15 kDa-170 kDa for the *in vitro* methylation reaction of the EF-Tu depleted lysate (D5) (Fig. 5B). This result implies that EF-Tu is the sole substrate for EftM. Re-addition of recombinant EF-Tu to the depleted lysate resulted in the reemergence of a single ~40 kDa band (Fig. 5B). Together, these results suggest that any physiological impact the presence of EftM has on the cell can be attributed exclusively to trimethylation of EF-Tu, and not modification of other proteins.

### Trimethylation of EF-Tu by EftM has little impact on the cellular steady-state proteome

While other post-translational modifications can impact EF-Tu’s canonical role in translation^13^ the K5me^3^ modification does not appear to alter global translation, as evidenced by similar total cellular protein and overall bacterial growth rate for PAO1 and PAO1∆*eftM*^15^. Therefore we hypothesized that if this particular post-translational modification impacted EF-Tu canonical function, the result would be altered abundance of specific proteins and not altered translation rate in general. Such an influence on translation would be similar, for example, to how EF-P increases translation of proteins with polyproline stretches^20^. To test this idea we compared the entire proteome in an unbiased, label-free method between the wild-type strain, a strain in which *eftM* was inactivated, and a complemented mutant that restores *eftM* activity to native levels.

For our standard laboratory strain PAO1 and its derivatives, we grew strains at 25 °C until mid-exponential phase. This is the temperature at which EftM is stable and active, and also the temperature at which we observed additional up-regulation at the level of transcription. Mid-exponential phase was assessed because that is the condition under which EF-Tu is most abundant and active, thus maximizing any potential differences in translation due to methylation status. To ensure reliable and reproducible insights, we performed a full comparison in PAO1 two independent times, named Data Set #1 and Data Set #2 (Supplemental Table 1). In each data set, the wild-type PAO1 (WT), Δ*eftM* deletion (∆), and PAO1-complemented (Comp) strains were grown and analyzed in independent biological triplicates.

**Figure 6:**
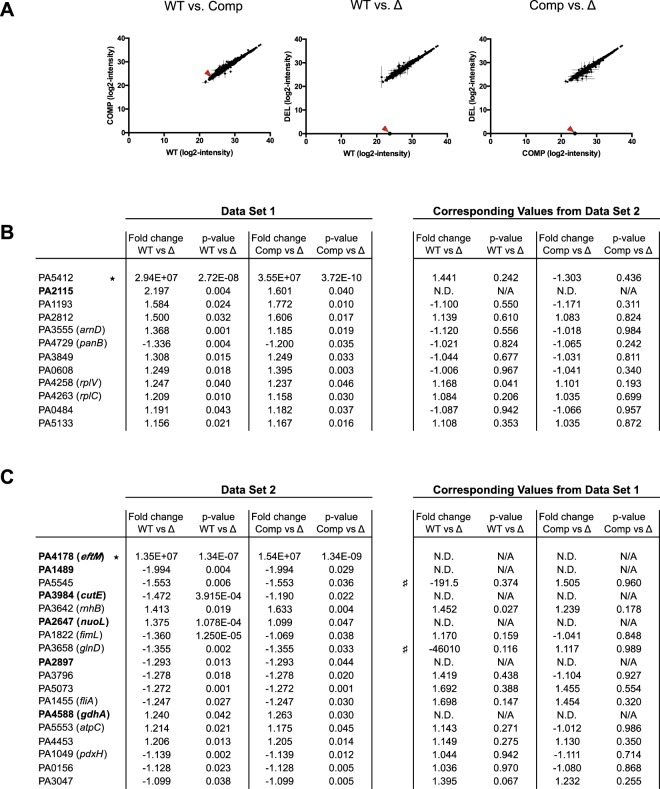
Whole-cell proteomic analysis reveals that trimethylation of EF-Tu by EftM has little impact on the proteome. (**A**) Plot of the log2-transformed label-free quantification (LFQ) intensities for all proteins detected in PAO1 Data Set 2. EftM is denoted by the red arrows. (**B**) Proteins from PAO1 Data Set 1 significantly changing in Δ (PAO1*Δeft*M) compared to WT (PAO1) and Comp (PAO1*ΔeftM at*tTn7::*PeftM*-_*eftM*_-FLAG). The corresponding values from Data Set 2 are listed to the right for comparison. (**C**) Proteins from Data Set 2 significantly changing in Δ (PAO1*Δeft*M) compared to WT (PAO1) and Comp (PAO1*ΔeftM at*tTn7::*PeftM*-_*eftM*_-FLAG). The corresponding values from Data Set 1 are listed to the right for comparison. Bold; proteins detected in one data set only. Positive fold change; abundance is higher in strain with EftM. Negative fold change; abundance is higher in strain without EftM. ^★^Denotes that the protein was not detected in all three biological triplicates of Δ. ^♯^Denotes not detected in biological triplicates of WT. N.D; not detected in all three biological triplicates of all three strains (WT, Δ, Comp) for that data set. N/A; not applicable.

Data were first examined by plotting detection intensity (label-free quantitation; LFQ) for each individual protein as comparisons between strains. WT and Comp showed a 1:1 trend line, indicating that these two strains were well matched (Fig. 6A, left). We plotted the same for WT vs. Δ and Comp vs. Δ to identify outliers in protein abundance (Fig. 6A, center and right). EftM was immediately apparent as different between the strains with and without EftM (red arrows), but other obvious significant differences were not immediately apparent.

To examine the data in more depth, data sets were analyzed for proteins significantly changing between WT and Δ using a *p*-value of 0.05 as the cut-off for significance, while no minimum for fold-change was imposed. These values were chosen in line with our goal of identifying any potential differences for further exploration, even those that are weak. The same analysis was then performed comparing Comp to Δ, and the two lists cross-referenced for those proteins that showed overlap with fold-changes in the same direction (i.e. both higher in the strain with EftM or both higher in the strain without EftM). This final list of significantly changing proteins consisted of 12 proteins for Data Set #1, and 18 proteins for Data Set #2 (Fig. 6B). Ultimately no proteins overlapped and were deemed significant from both Data Sets, as evaluated by changes in protein abundance with *p* < 0.05. Combining the lists yielded from Data Set #1 and Data Set #2 resulted in 30 candidate proteins. Twenty-three proteins were detected in both analyses, but only showed significance in one set. Six proteins showed significance in one set but were not detected (below the limit of detection) in the alternate set, meaning they cannot be strictly eliminated as proteins potentially impacted by EF-Tu lysine 5 trimethylation (Fig. 6B,C, bold). The remaining protein was EftM, which despite being very lowly expressed, was detected in Data Set #2 as being present in the wild-type and complement and absent in the deletion strain, with a *p*-value of 1.33 × 10^−7^. This result gave us additional confidence in the quality and accuracy of our analysis.

In addition to PAO1, strain PA14 was also analyzed for global proteome changes to eliminate potential strain bias in our conclusions. Again, the analysis of the PA14 strains (WT, ∆, Comp) was performed in biological triplicate at 25 °C at mid-exponential phase. We observed just two proteins that were statistically significant. One was due to an absence of detection of the protein in all three biological replicates of Δ (Supplemental Table 2). This protein, PA14_24370, is a homolog of PA3076 and was detected in all three strains of the PAO1 Data Set #2 series with no significant changes between strains. The other is PA14_64050, which was detected in both PAO1 Data Set #1 and Data Set #2 with no significant changes.

Lastly we took advantage of our clinical isolate PAHM4, which contains a version of EftM with structural stability at elevated temperature, and performed the same comparisons between the proteomes of these strains (WT, ∆, and Comp). For this analysis the strains were grown at 37 °C instead of 25 °C. Elevated growth temperature allowed us to capture data on any proteins that would have not been expressed in our 25 °C analysis. In addition to temperature elevation, the PAHM4 data set is different in that the PAHM4 genome has 377,121 bp of DNA not found in our other two analyzed strains^18^. PAHM4 also expresses several gene clusters not normally translated in our standard laboratory reference strains, including those conferring a mucoid phenotype. Overall this allowed us to examine a widely different pool of expressed proteins to detect anything that would have been missed with our initial experimental conditions. Of the 10 candidate proteins derived from this strain comparison, the analysis of PAHM4 ultimately yielded the same conclusion (Supplemental Table 3) as for PAO1 and PA14. One protein (PAHM4_RS15825) was detected as being more abundant in the deletion strain but is encoded in a region of the genome that has no PAO1 or PA14 equivalent, meaning there is no way to compare this finding with other data sets. One (PAHM4_RS17805) is a homolog of PA2912, but this protein was not detected in PAO1 or PA14. The other eight proteins identified as significantly changing in PAHM4 were detected in at least one data set of PAO1 and PA14, where they were confidently determined to be not significant.

## Discussion

*P*. *aeruginosa* is an opportunistic pathogen that can survive and thrive in a wide variety of environments and temperatures, including those which are terrestrial, freshwater, or marine ranging from 4 °C to 42 °C, as well as diverse hosts such as plants, worms, and mammals^21,22^. This wide range of inhabitable environments requires a flexible repertoire of gene expression to customize the proteomic output of the bacterium to the appropriate environment. Temperature sensing is one way in which to accomplish this goal. With over 6.26 million base pairs and 5,570 open reading frames in PAO1^23^, timing of gene expression in the appropriate context is an important aspect of bacterial pathogenesis.

Temperature-mediated regulation of protein expression can be achieved by the cell in a variety of manners. One point of regulation can be transcriptional, through the use of temperature-sensitive transcription factors. Post-transcriptional control can be attained through RNA secondary structure, most commonly termed RNA thermometers. Post-translational control is accomplished through selective degradation of proteins with temperature-responsive proteases or inherent temperature sensitivity of the protein.

While not common, some proteins are thermoregulated at two levels of production, or “dual-thermoregulated”. One example is LcrF, a global regulator in *Yersinia pseudotuberculosis* that is thermoregulated at both the translational and transcriptional level. The 5′UTR of *lcrF* mRNA has an RNA thermometer, while transcription is repressed by nucleoid-like protein YmoA, a protein which is selectively degraded at 37 °C^24^.

We previously reported that EftM is intrinsically thermo-unstable, meaning at the down-regulated temperature (37 °C), the enzyme itself was unfolded and inactive. Therefore EftM can be considered a “protein thermometer” that directly senses and responds to elevated temperature. Here we have uncovered a second layer of regulation for this protein, making it a dual thermoregulated methyltransferase. Not only does EftM itself unfold at 37 °C, but transcription of the mRNA encoding this methyltransferase is also down-regulated, demonstrating an intriguing second level of thermoregulation. We have uncovered evidence that suggests this second layer of thermoregulation occurs during transcription initiation, as evidenced by β-galactosidase assay data with *eftM* reporter fusion strains presented in Fig. 4. An obvious potential mechanism for this phenotype could be a temperature-responsive transcription factor that either promotes transcription at 25 °C or represses transcription at 37 °C. Secondary structure of the DNA could also be a factor in accessibility, although this form of regulation is typically observed when 37 °C is the permissive temperature. Nonetheless, secondary structure may play a role when in combination with histone-like structuring proteins which could, in theory, no longer recognize the *eftM* promoter at 25 °C, thus alleviating repression. Lastly, while unlikely, it is possible that transcription initiation is the same at both temperatures; instead, unpredicted secondary structure of the 5′UTR revealed in Fig. 3 could be impacted by temperature. At the higher temperature, partial unmelting of mRNA secondary structure could increase the accessibility to RNase sites leading the degradation of the transcript. Work is currently underway to reveal the exact mechanism mediating this intriguing transcriptional thermoregulation.

EftM being subject to dual thermoregulation is curious, as levels of *eftM* transcript in the cell are intrinsically relatively low. In fact, in three different studies of temperature regulation, *eftM* was undetectable at either temperature by whole-cell transcriptome profiling^2,5,9^. Down-regulation at 37 °C further lowers these transcript levels in a seemingly redundant attenuation mechanism. It is interesting to note that in our hypermutator clinical isolate PAHM4, protein stability is enhanced, while transcriptional thermoregulation is relatively unaltered, leaving only one layer of thermoregulation in this particular strain for the shift from ambient to physiological temperature.

Since EftM trimethylates EF-Tu, the elongation factor that delivers charged tRNAs to the translating ribosome, we were interested in how this modulation would affect protein synthesis. However, we first needed to confirm that EF-Tu was the only substrate for EftM methylation. If there were other substrates or interactions, we would not be able to attribute any phenotype observed exclusively to EF-Tu trimethylation.

Our typical read-out for EftM activity is Western blot with a trimethyl lysine antibody. This works extremely well for detecting trimethylation of EF-Tu, as we observe no bands in whole cell lysates of an *eftM*-deleted strain, and one distinct band at the size of EF-Tu for strains containing active EftM. This method of detection presents problems for identifying substrates outside of EF-Tu, however. Therefore to test whether EftM modified other substrates we utilized an *in vitro* methylation assay that included EftM, radiolabeled SAM, and EF-Tu depleted *P*. *aeruginosa* lysates. Using a radiolabeled SAM on an EF-Tu depleted lysate was important for two reasons. First, observation of tritiated methyl transfer is non-specific in its readout, whereas an antibody is specific to trimethylation with reactivity that can be influenced by structural context. Secondly, EF-Tu can be over 10% of the total cellular protein in exponential phase of growth. Given that EF-Tu is a known substrate for EftM, the signal from EF-Tu has the potential to overshadow the signal from any other substrates. By eliminating EF-Tu in our whole-cell lysates through immunoprecipitation we were able to more sensitively see any other potential methyl-transfer event. After successfully depleting both 25 °C and 37 °C lysates for EF-Tu, we performed an *in vitro* methylation reaction with radio-labeled SAM and recombinant purified EftM. We observed no substrates for EftM. We then re-added EF-Tu to our reaction conditions and observed methyl transfer, showing that our reaction conditions were appropriate and our purified EftM was active. Overall we can conclude that EftM’s only observable substrate for cellular methylation is EF-Tu.

This information allowed us to undertake a large-scale proteomic analysis of three distinct *P*. *aeruginosa* isolates to assess the impact that EftM-mediated EF-Tu trimethylation has on EF-Tu’s canonical role in protein translation. With the proteomes of three strains and derivatives analyzed in biological triplicate, in an unbiased, label-free method, we observed no significant and reproducible differences in protein levels dependent on EF-Tu K5me^3^ status. We chose to use mid-exponential phase of growth as our standard for our study given that EF-Tu is most prominent and active in translation during these conditions. A growth temperature of 25 °C was chosen for PAO1 and PA14 because that is the temperature under which we see multiple layers of up-regulation in EftM expression, as reported previously^16^ and in this current study. We also chose to analyze the PAHM4 proteome at 37 °C to take advantage of our strain with increased EftM stability. Including this strain in our study widened our pan-proteome analysis to proteins beyond just those expressed in our laboratory strains, giving us confidence that the effect of EF-Tu trimethylation on canonical function was assessed with a wide variety of protein templates.

Out of the over 5,000 open-reading frames in *P*. *aeruginosa* PAO1 genome, we detected 2,327 unique proteins in Data Set #1 and 2,648 unique proteins in Data Set #2, with a total of 2,733 proteins observed between the two. We anticipated that, were EF-Tu K5me^3^ impacting translation, we would see a change in abundance in a set of these expressed proteins. Yet from these candidates we have found a very small number, only eight, which we could not strictly eliminate as potentially changing in response to EftM presence. These eight seemingly unrelated proteins (PA2115, PA1489, PA3984, PA2647, PA2897, PA4588; Fig. 6. PAHM4_RS15825, PAHM4_RS17805, Supplemental Table 3) were determined to be significantly changing with a *p*-value < 0.05. Therefore, these eight proteins are potentially differentially translated in response to EF-Tu lysine 5 methylation status. However, due to the modest fold-changes observed (1.085 → 2.197 fold), the small number of final candidates with no apparent connections or similarities in terms of function, chromosomal location, or predicted subcellular location, and the fact that they were only detected in individual data sets, we ultimately conclude that there is little alteration of EF-Tu’s canonical function in *P*. *aeruginosa* in response to K5 methylation status. It is possible that EF-Tu K5me^3^ has impact under other conditions not tested, such as during stationary phase or nutrient limitation, or only alters lowly expressed proteins which are thus under the limit of detection of our instrumentation. Additionally, cellular pellets were washed to remove traces of growth media that would interfere with mass-spectrometry analysis. This processing removes secreted proteins from our samples. Despite these caveats, our investigation has provided the first analysis of EF-Tu K5me^3^’s impact on translation and has demonstrated no widespread effect of EftM on protein production.

Given that EftM does not broadly impact the proteome of *P*. *aeruginosa* through post-translational modification of EF-Tu, the previously-defined role of K5me^3^ EF-Tu as an adhesin to platelet-activating factor receptor-containing epithelial cells^15^ may be more important than previously appreciated, especially given the wide degree of conservation of EftM throughout the *Pseudomonads* and *Vibrio* genus. Dual thermoregulation also hints to the importance of maintaining an appropriate level of EftM in the cell. EftM may be beneficial in the ambient environment or during the initial stages of infection, such as for adherence to epithelial cells. However, upon sensing a shift to 37 °C, *P*. *aeruginosa* not only stops transcribing and therefore translating new EftM, but also rapidly inactivates the current pool of EftM through unfolding of the existing protein. There is no known demethylase associated with EftM, suggesting that the pool of methylated EF-Tu is all that remains of EftM’s impact on the cell. This most likely takes several generations to dilute out, giving *P*. *aeruginosa* a gradual transition to unmethylated EF-Tu – in stark contrast to the immediate and redundant shut-off of EftM. PAHM4 has a form of EftM with increased thermostability. It will be interesting to elucidate the benefit to this particular strain gaining a thermostable form of EftM. It may be a benefit during chronic non-cystic fibrosis bronchiectasis, the infection from which this isolate was gathered, or there may be compensatory mutations elsewhere in the genome that eliminate the detriment of thermostable EftM. Further investigation into this strain may reveal a widened scope to EftM function in the cell.

## Methods

### Strains

PAO1, PA14, and PAHM4 with derivatives are described in Supplemental Table 4. PAO1Δ*eftM*, previously described, was created by deletion of 100 bp within the coding sequence of *eftM*^15^. PA14_08970::MAR2xT7 (PA14 *eftM*::tn) is a transposon mutant from the PA14 library^25^. PAHM4Δ*eftM* was made using the same general strategy as PAO1Δ*eftM* with two major differences: first, selection for double recombinants was performed with gentamicin 400 μg/ml instead of 30 μg/ml, and second, the gentamicin cassette was not subsequently removed.

Complementation strains were constructed using the Tn7 system for site-specific integration into the *P*. *aeruginosa* chromosome^26^. Briefly, 500 bp upstream of the coding sequence for *eftM*, as well as the entire *eftM* coding sequence, were cloned from PAO1 (oligos SMP10 + SMP45; Supplemental Table 5) or PAHM4 genomic DNA (oligos SMP17 + SMP47; Supplemental Table 5) with the addition of a FLAG-tag at the C-terminal end of the protein. After PCR fragments were ligated into pUC18T-miniTn7T-Tp, the resulting construct was inserted in single copy at the Tn7 attachment site by electroporation along with the helper plasmid pTNS3 into PAO1∆*eftM* or PAHM4∆*eftM*. Reporter strains for β-galactosidase assays were constructed using the CTX system for single-copy delivery to the *Pseudomonas* genome^19^ with subsequent FRT-mediated excision of the plasmid backbone using pFLP2. The *rpoD* reporter plasmid was created by amplifying P_*rpoD*_ with primers SMP176 + SMP177 and *lacZ* with SMP178 + SMP179; the *eftM* reporter plasmid was created by amplifying P_*eftM*_ with SMP189 + SMP196 and *lacZ* with SMP179 + SMP184. Fragments were seamlessly fused with the CTX plasmid using isothermal assembly to create translational fusions. All reporters were inserted into PAO1 through electroporation. A promoterless *lacZ* was inserted as a negative control. All plasmid constructs described were confirmed by Sanger sequencing and integrations confirmed through PCR.

### Western immunoblots

*P*. *aeruginosa* was grown at indicated temperature in LB with rotation until mid-exponential phase (OD_600_ = 0.8). Cultures were standardized such that an amount of cells equivalent to 1 mL of an OD_600_ = 1 culture were harvested by centrifugation, then the cell pellet was resuspended in 100 μl Laemmli buffer. Suspensions were boiled for 5 minutes, 10 μl loaded into a 10% polyacrylamide gel, and samples were separated by SDS-PAGE. After transfer to PVDF, membranes were blocked and incubated in αTrimethyl Lysine (ImmuneChem ICP0601, blocked with 5% BSA), αEF-Tu (Hycult mAb 900, blocked with 5% skim milk), or αRpoA (Neoclone, blocked with 5% skim milk) at 4 °C overnight with rocking. Next membranes were incubated with appropriate secondary antibody for one hour at room temperature, exposed with Pierce ECL Western Blotting Substrate (ThermoFisher), and imaged on a ChemiDoc MP Imaging System.

### Depletion of EF-Tu from *P*. *aeruginosa* lysates and *in vitro* methylation

PAO1Δ*eftM* was grown to mid-exponential at 25 °C or 37 °C. Cells were lysed by sonication and depleted 5 times by incubating lysate with an α-EF-Tu antibody (Hycult Biotech). The complex was then immobilized by magnetic Protein G Dynabeads (ThermoFisher) and a magnet while the rest of the lysate was transferred to a fresh tube. Lysate was collected after each round and analyzed by immunoblot using α-EF-Tu or α-RpoA (Neoclone; loading control). For the *in vitro* methylation reaction, 20 μg of the undepleted or final depleted lysate was incubated with 3.6 μg purified EftM (8 μM final concentration) and [^3^H]-SAM at 25 °C for 20 minutes, then separated by SDS-PAGE. The gel was dried, exposed to a tritium screen overnight, and scanned using a Typhoon imager. Recombinant *P*. *aeruginosa* EF-Tu (rEF-Tu) purified from *E*. *coli*^16^ was added to final depleted lysate as a control and to determine the limit of detection.

### RNA isolation and RT-qPCR

Strains were grown at the indicated temperature in LB with rotation until exponential phase was achieved (OD_600_ = 0.8). After harvesting, cells were lysed and nucleic acid isolated with a MasterPure RNA Purification Kit (Epicentre) according to manufacturer’s instructions. Residual contaminating DNA was removed with TURBO DNase (ThermoFisher) and isolated RNA was converted to cDNA with TaqMan Reverse Transription reagents (Invitrogen) prior to analysis with FastStart Universal SYBR Green Master (Rox; Roche) on a Roche LightCycler 96. Primers SMP155 + SMP156 were utilized for *eftM;* oJV1040 + oJV1041 for *omlA*; and *rpoD* F + *rpoD* R for *rpoD*^17^ (Supplemental Table 5). Significance was determined by two-way ANOVA with Sidak multiple comparisons analysis (GraphPad Prism version 6.0).

### 5′ Rapid amplification of cDNA ends (5′RACE)

mRNA was isolated as described above from strain PAO1 and PAHM4 grown at both 25 °C and 37 °C. 5′RACE was performed as per manufacturer’s instructions using the 5′ RACE System for Rapid Amplification of cDNA Ends, version 2.0 (Thermo Fisher). Briefly mRNA was converted to cDNA with Gene Specific Primer 1 (GSP1), 3′ end of the cDNA tailed with a poly-C tail, and the resulting cDNA amplified with primer GSP2 before Sanger sequencing for the junction between the 5′ untranslated region (UTR) and the poly-C tail. GSPs utilized for reverse transcription and nested amplification are SMP22 and SMP49 (Supplemental Table 5). Transcription start site determination allowed for promoter prediction using Virtual Footprint Version 3.0^27^.

### β-galactosidase assays

Reporter strains were grown in LB at 25 °C or 37 °C to mid-exponential phase prior to collection for the assay. After centrifugation, pellets were resuspended in 1 mL Z buffer (60 mM sodium phosphate dibasic, 40 mM sodium phosphate monobasic, 10 mM potassium chloride, 1 mM magnesium sulfate, 50 mM β-mercaptoethanol, pH 7.0). Sample aliquots were added to Z buffer to a total volume of 1 mL (200 μl of P_*rpoD*_ was added to 800 μl Z buffer; 900 μl P_*eftM*_ to 100 μl Z buffer). Then 100 μl chloroform 50 μl 0.1% SDS were added to each sample, vortexed, and samples incubated at 30 °C. After addition of 200 μl of 4 mg/mL ortho-nitrophenyl-β-galactoside, reactions were timed until the development of a yellow color, at which point 400 μl of 1 M sodium carbonate was added to terminate the reaction. Cell debris was removed by centrifugation and the sample supernatant was read at 420 nm. Miller units were calculated as follows: (1,000 × A_420_)/(reaction time in minutes × cell suspension volume in mL × OD_600_). Significance was determined by two-way ANOVA with Sidak multiple comparisons analysis (GraphPad Prism version 6.0).

### Proteomic sample preparation

*P. aeruginosa* was grown overnight in liquid culture with aeration at 25 °C (for PAO1 derivatives and PA14 derivatives) or 37 °C (for PAHM4 derivatives). After back-dilution to OD_600_ = 0.05, cells were allowed to grow until exponential phase was achieved (OD_600_ = 0.8). Cells were harvested by centrifugation and washed with sterile PBS before re-pelleting and storage at −80 °C until analysis. After thawing, the cell pellet was lysed in 200 µL of urea lysis buffer (8 M urea, 100 mM NaHPO_4_, pH 8.5), including 2 µL (100x stock) HALT protease and phosphatase inhibitor cocktail (Pierce). Protein supernatants were transferred to new 1.5 mL Eppendorf tubes and sonicated (Sonic Dismembrator, Fisher Scientific) 3 times for 5 seconds with 15 second intervals of rest at 30% amplitude to disrupt nucleic acids and subsequently vortexed. Protein concentration was determined by the bicinchoninic acid (BCA) method, and samples were frozen in aliquots at −80 °C. Protein homogenates equivalent to 100 μg of total protein were then treated with 1 mM dithiothreitol (DTT) at 25 °C for 30 minutes, followed by 5 mM iodoacetimide (IAA) at 25 °C for 30 minutes in the dark. The lysate was next digested with 1:100 (w/w) lysyl endopeptidase (Wako) at 25 °C overnight. The samples were then diluted with 50 mM NH_4_HCO_3_ to a final concentration of ~1 M urea and further digested overnight with 1:50 (w/w) trypsin (Promega) at 25 °C. Resulting peptides were desalted with a Sep-Pak C18 column (Waters) and dried under vacuum.

### LC-MS/MS analysis

Dried peptides were resuspended in 100 µL of peptide loading buffer (0.1% formic acid, 0.03% trifluoroacetic acid, 1% acetonitrile). Peptide mixtures (2 μL) were separated on a self-packed C18 (1.9 μm Dr. Maisch, Germany) fused silica column (25 cm × 75 μM internal diameter (ID); New Objective, Woburn, MA) by a Dionex Ultimate 3000 RSLCNano and monitored on a Fusion mass spectrometer (ThermoFisher Scientific, San Jose, CA). Elution was performed over a 120-minute gradient at a rate of 300 nl/minute with buffer B ranging from 3% to 80% (buffer A: 0.1% formic acid in water, buffer B: 0.1% formic in acetonitrile). The mass spectrometer cycle was programmed to collect at the top speed for 3-second cycles. The MS scans (400–1600 m/z range, 200,000 AGC, 50 ms maximum ion time) were collected at a resolution of 120,000 at m/z 200 in profile mode and the HCD MS/MS spectra (0.7 m/z isolation width, 30% collision energy, 10,000 AGC target, 35 ms maximum ion time) were detected in the ion trap. Dynamic exclusion was set to exclude previous sequenced precursor ions for 20 seconds within a 10 ppm window. Precursor ions with +1 and +8 or higher charge states were excluded from sequencing.

### Proteomic data analysis

RAW data for the samples was analyzed using MaxQuant v1.5.2.8 with Thermo Foundation 2.0 for RAW file reading capability (Supplemental Table 1). The search engine Andromeda, integrated into MaxQuant, was used to search strain-appropriate *Pseudomonas* protein databases (Downloaded from pseudomonas.com^28^), plus 245 contaminant proteins from the common repository of adventitious proteins (cRAP) built into MaxQuant. Methionine oxidation (+15.9949 Da), asparagine and glutamine deamidation (+0.9840 Da), and protein N-terminal acetylation (+42.0106 Da) were variable modifications (up to 5 allowed per peptide); cysteine was assigned a fixed carbamidomethyl modification (+57.0215 Da). Only fully tryptic peptides were considered with up to 2 miscleavages in the database search. A precursor mass tolerance of ±20 ppm was applied prior to mass accuracy calibration and ±4.5 ppm after internal MaxQuant calibration. The match between runs option was used and quantification was based on final normalized LFQ outputs.

For both PAO1 Data Set 1 and Data Set 2, wild-type (WT), deletion (Δ), and complement (Comp) strains were grown and analyzed in biological triplicate. Signal was log_2_-transformed and averaged across the biological triplicates, with p-values determined within the biological triplicates by ANOVA with Tukey post-hoc pairwise comparison or two tailed *t*-test when ANOVA was not possible. Results were filtered for proteins in which *p* < 0.05 for both WT vs. Δ and Comp vs. Δ with fold changes occurring in the same direction. The same was repeated with PA14 strains and PAHM4 strains.

## Supplementary information

Supplemental Table 1

Supplemental Tables 2–5
