# Supplementary material for: Trimethylation of Elongation Factor-Tu by the Dual Thermoregulated Methyltransferase EftM Does Not Impact Its Canonical Function in Translation
Source: Sci Rep. 2019 Mar 5;9:3553. doi: 10.1038/s41598-019-39331-x (PMC6401129; doi:10.1038/s41598-019-39331-x)
Supplement: Supplementary file 2 — Supplemental Tables 2–5 [file 41598_2019_39331_MOESM2_ESM.pdf]

Trimethylation of Elongation Factor-Tu by the Dual Thermoregulated Methyltransferase EftM Does Not Impact Its Canonical Function in Translation

Samantha M. Prezioso[1,2], Duc M. Duong[3], Emily G. Kuiper[4,5,], Qiudong Deng[3], Sebastián Albertí[6], Graeme L. Conn[5,7], Joanna B. Goldberg[2,7,8*]

*Correspondence and requests for materials should be addressed to JBG (email: joanna.goldberg@emory.edu)

Affiliations

[1]Microbiology and Molecular Genetics (MMG) Program, Graduate Division of Biological and Biomedical Sciences, Emory University, Atlanta, GA 30322, USA.
[2]Division of Pulmonology, Allergy/Immunology, Cystic Fibrosis and Sleep, Department of Pediatrics, Emory University School of Medicine, Atlanta, GA 30322, USA.
[3]Emory Integrated Proteomics Core, Emory University, Atlanta GA 30322
[4]Biochemistry, Cell and Developmental Biology (BCDB) Program, Graduate Division of Biological and Biomedical Sciences, Emory University, Atlanta, GA 30322, USA.
[5]Department of Biochemistry, Emory University School of Medicine, Atlanta, GA 30322, USA.
[6]Instituto Universitario de Investigación en Ciencias de la Salud, Universidad de las Islas Baleares, Palma de Mallorca, Spain.
[7]Emory Antibiotic Resistance Center, Atlanta, GA 30322, USA.
[8]Emory+Children's Center for Cystic Fibrosis and Airway Disease Research, Atlanta, GA 30322, USA.

Supplemental Table 2

		Significant Proteins from PA14				Corresponding Values from PAO1 Data Set 1				Corresponding Values from PAO1 Data Set 2				Corresponding Values from PAHM4			
		**Fold change WT vs Δ**	**p-value WT vs Δ**	**Fold change Comp vs Δ**	**p-value Comp vs Δ**	Fold change WT vs Δ	p-value WT vs Δ	Fold change Comp vs Δ	p-value Comp vs Δ	Fold change WT vs Δ	p-value WT vs Δ	Fold change Comp vs Δ	p-value Comp vs Δ	Fold change WT vs Δ	p-value WT vs Δ	Fold change Comp vs Δ	p-value Comp vs Δ
**PA14_24370**	⋆	**1.3E+07**	**4.2E-10**	**1.1E+07**	**3.1E-11**	N.D.	N/A	N.D.	N/A	-1.298	0.367	-1.094	0.870	-1.212	0.345	-1.288	0.190
**PA14_64050**	♯	**-1.3E+05**	**0.003**	**-1.283**	**0.019**	1.066	0.985	1.037	0.991	-1.680	0.056	-1.467	0.151	N.D.	N/A	N.D.	N/A

Supplemental Table 3

		Significant Proteins from PAHM4				Corresponding Values from PAO1 Data Set 1				Corresponding Values from PAO1 Data Set 2				Corresponding Values from PA14			
		**Fold change WT vs Δ**	**p-value WT vs Δ**	**Fold change Comp vs Δ**	**p-value Comp vs Δ**	Fold change WT vs Δ	p-value WT vs Δ	Fold change Comp vs Δ	p-value Comp vs Δ	Fold change WT vs Δ	p-value WT vs Δ	Fold change Comp vs Δ	p-value Comp vs Δ	Fold change WT vs Δ	p-value WT vs Δ	Fold change Comp vs Δ	p-value Comp vs Δ
**PAHM4_RS15825**	N/A	**-1.647**	**0.015**	**-1.668**	**0.014**	N/A	N/A	N/A	N/A	N/A	N/A	N/A	N/A	N/A	N/A	N/A	N/A
**PAHM4_RS08095**	(PA5074)	**-1.480**	**2.914E-04**	**-1.153**	**0.044**	1.967	0.083	1.588	0.241	-1.164	0.343	-1.083	0.713	1.088	0.919	-1.072	0.949
**PAHM4_RS12930**	(PA3712)	**1.457**	**0.012**	**1.476**	**0.010**	1.179	0.642	1.066	0.932	-1.255	0.091	-1.085	0.645	1.242	0.497	-1.139	0.758
**PAHM4_RS25435**	(PA3106)	**-1.394**	**0.030**	**-1.363**	**0.039**	-1.191	0.705	-1.025	0.993	-1.106	0.430	1.077	0.609	-1.057	0.928	1.129	0.716
**PAHM4_RS17805**	(PA2912)	**-1.348**	**4.795E-06**	**-1.085**	**0.007**	N.D.	N/A	N.D.	N/A	N.D.	N/A	N.D.	N/A	N.D.	N/A	N.D.	N/A
**PAHM4_RS18780**	(PA1117)	**-1.330**	**0.020**	**-1.336**	**0.018**	1.344	0.640	1.651	0.395	-1.031	0.980	1.094	0.841	♯ -319.367	0.837	1.013	0.999
**PAHM4_RS16925**	(PA0354)	**-1.242**	**0.001**	**-1.133**	**0.015**	1.053	0.872	-1.250	0.152	1.000	1.000	1.010	0.979	-1.076	0.832	1.014	0.993
**PAHM4_RS10405**	(PA1880)	**-1.235**	**0.021**	**-1.287**	**0.009**	N.D.	N/A	N.D.	N/A	-1.070	0.926	-1.189	0.770	1.091	0.868	-1.097	0.851
**PAHM4_RS00050**	(PA0225)	**-1.138**	**0.042**	**-1.137**	**0.044**	N.D.	N/A	N.D.	N/A	-1.347	0.480	1.139	0.856	N.D.	N/A	N.D.	N/A
**PAHM4_RS05090**	(PA4329)	**1.098**	**0.001**	**1.052**	**0.024**	1.045	0.781	1.089	0.437	1.046	0.658	-1.058	0.535	1.142	0.461	-1.046	0.902

Supplemental Table 2: Whole-cell proteomic analysis reveals that trimethylation of EF-Tu by EftM has little impact on the proteome.
Proteins from PA14 significantly changing in Δ (PA14 *eftM*::tn) compared to WT (PA14) and Comp (PA14 *eftM::*tn *att*Tn7::P_{eftM}-*eftM*-FLAG). The corresponding values from PAO1 and PAHM4 are listed to the right for comparison.

Bold; proteins detected in one data set only. Positive fold change; abundance is higher in strain with EftM. Negative fold change; abundance is higher in strain without EftM. ⋆ denotes that the protein was not detected in all three biological triplicates of Δ; ♯ denotes not detected in biological triplicates of WT. N.D; not detected in all three biological triplicates of all three strains (WT, Δ, Comp) for that data set. N/A; not applicable.

Supplemental Table 3: Whole-cell proteomic analysis reveals that trimethylation of EF-Tu by EftM has little impact on the proteome.
Proteins from PAHM4 significantly changing in Δ (PAHM4Δ*eftM*) compared to WT (PAHM4) and Comp (PAHM4Δ*eftM* *att*Tn7::$P_{eftM\text{-}HM4}$-$eftM_{HM4}$-FLAG). The corresponding values from PAO1 and PA14 are listed to the right for comparison.

Bold; proteins detected in one data set only. Positive fold change; abundance is higher in strain with EftM. Negative fold change; abundance is higher in strain without EftM. ⋆ denotes that the protein was not detected in all three biological triplicates of Δ; ♯ denotes not detected in biological triplicates of WT. N.D; not detected in all three biological triplicates of all three strains (WT, Δ, Comp) for that data set. N/A; not applicable.

Supplemental Table 4. Strain List

ID	Genotype	Reference
PAO1	*P. aeruginosa* PAO1	Barbier, Owings *et al* 2013
PAO1Δ*eftM*	PAO1 *eftM* Δ255-487	Barbier, Owings *et al* 2013
PASP09	PAO1ΔeftM *att*Tn7::P_{eftM}-*eftM*$_{PAO1}$-FLAG	This Study
PASP106	PAO1ΔeftM *att*Tn7::$P_{eftM\text{-}10SCR}$-*eftM*$_{PAO1}$-FLAG	This Study
PASP20	PAO1ΔeftM *att*Tn7::P_{eftM}-*eftM*$_{HM4}$-FLAG	This Study
PASP76	PAO1 *att*CTX::P_{eftM}-*lacZ FRT*	This Study
PASP65	PAO1 *att*CTX::P_{rpoD}-*lacZ FRT*	This Study
PA14	*P. aeruginosa* PA14	Rahme, Stevens *et al.* 1995
PA14 *eftM*::tn	PA14_08970::MAR2xT7	Liberati *et al.* 2006
PASP12	PA14 *eftM::*tn *att*Tn7::P_{eftM}-*eftM*$_{PAO1}$-FLAG	This Study
PAHM4	*P. aeruginosa* PAHM4	Varga *et al.* 2015
PAHM4Δ*eftM*	PAHM4 *eftM*Δ255-487	This Study
PASP51	PAHM4Δ*eftM att*Tn7::P_{eftM}-*eftM*$_{HM4}$-FLAG	This Study

Supplemental Table 5. Oligonucleotides

ID	Target	Sequence
SMP10	complementation of *eftM* (PAO1)	TGG ATC CCC AGA CCT TCC ACG GCA GTT G
SMP45	complementation of *eftM* (PAO1) with FLAG tag	TGA ATT CCT ACT TGT CAT CGT CAT CCT TGT AGT CGC GCT TCA CGC AGA C
SMP216	creation of scrambled -10 promoter	AAG GCC CGG GCC TGG GGA TTA CCG CCG CGC CCT TCG CCG CCG GTC C
SMP217	creation of scrambled -10 promoter	CAG GTG CTG CAG CTC GGC GAA
SMP17	complementation of eftM (PAHM4)	TGG ATC CAA CCA GAT CCT CCA CGG CAG TTG
SMP47	complementation of eftM (PAHM4) with FLAG tag	TGA ATT CCT ACT TGT CAT CGT CAT CCT TGT AGT CGC GCT TGA TGC AGG C
SMP189	P_{eftM}-*lacZ* reporter	TGG ATC CCC CGG GCT GCA GGA GTC CGC CCG CGA TCC GCC GGC CGG T
SMP196	P_{eftM}-*lacZ* reporter	TCA TGG TCA TGG GCG GCA AGG ACC GGC GGC GAA GGG CGC
SMP184	P_{eftM}-*lacZ* reporter	TCC TTG CCG CCC ATG ACC ATG ATT ACG GAT TC
SMP179	P_{eftM}-*lacZ* and P_{rpoD}-*lacZ* reporter	CGA GGT CGA CGG TAT CGA TAA GCT TTA TTT TTG ACA CCA GAC C
SMP176	P_{rpoD}-*lacZ* reporter	TGG ATC CCC CGG GCT GCA GGC CTT GAA AAG CAG TTC TTC GAC
SMP177	P_{rpoD}-*lacZ* reporter	TCA TGG TCA TAA CAC CCT ATC CAC TGA AGG T
SMP178	P_{rpoD}-*lacZ* reporter	GGA TAG GGT GTT ATG ACC ATG ATT ACG GAT TC
SMP155	*eftM* qPCR	GAC ATC AAC CAG CCG ATG CTC
SMP156	*eftM* qPCR	CAG CCC TGC GTT GTA GTG GA
rpoD F	*rpoD* qPCR	GGG CGA AGA AGG AAA TGG TC
rpoD R	*rpoD* qPCR	CAG GTG GCG TAG GTG GAG AA
oJV1040	*omlA* qPCR	AAA ATC GAC ATC CAG CAA GG
oJV1041	*omlA* qPCR	GGT CGC TGT CGT TGA AGA AC
SMP22	*eftM* 5' RACE GSP1	TGC GTT GTA GTG GAT CGA ATA G
SMP49	*eftM* 5' RACE GSP2	GAG CAT CGG CTG GTT GAT GTC
